# Testing a single item screener to support family doctors in identifying patients with limited health literacy: convergent validity of the SILS and the HLS-EU-Q16

**DOI:** 10.1186/s12875-023-02112-7

**Published:** 2023-08-09

**Authors:** Stephanie Stock, Arim Shukri, Sibel Altin, Farah Nawabi, Daniele Civello, Marcus Redaèlli, Adrienne Alayli

**Affiliations:** 1 Cologne Institute for Health Economics and Clinical Epidemiology, University of Cologne, Faculty of Medicine and University Hospital Cologne, Gleueler Str. 176-178, 50935 Koeln, Germany; 2 AOK Rheinland/Hamburg, Kasernenstraße 61, 40213 Düsseldorf, Germany; 3 Clinic of General Pediatrics, Neonatology and Pediatric Cardiology, Medical Faculty, Unit of Health Services Research, University Hospital Düsseldorf and Heinrich-Heine-University Düsseldorf, Moorenstraße 5, 40225 Düsseldorf, Germany

**Keywords:** Health literacy, Screening, Validation, Family practice, Chronic disease

## Abstract

**Background:**

Low health literacy (HL) is associated with reduced disease self-management skills, worse health outcomes, an increased number of hospitalizations, more frequent use of the emergency room and less utilization of preventive services. To support patients with low HL it is crucial to identify affected patients. HL is a multidimensional construct, which covers different skills and abilities to make informed health decisions. Validated brief screening tools to assess health-literacy-related skills or abilities in primary care settings are currently not available in German. This study aimed to validate a single item screener developed in the US for the German primary care setting.

**Methods:**

Our study used cross-sectional data from a survey among mainly chronically ill patients (*n* = 346) conducted in family practices in the state of North Rhine-Westphalia. We explored the convergent validity between a single item literacy screener (SILS) and the HLS-EU-Q16. The SILS measures functional HL by asking patients about their need for help when reading information materials. The HLS-EU-Q16 is a multidimensional HL measure frequently used for research purposes in Germany. Associations between the two instruments were examined using Spearman’s correlations and regression analyses. The diagnostic performance of the SILS relative to the HLS-EU-Q16 was assessed using receiver operator curves (ROC).

**Results:**

The SILS had a statistically significant correlation with the HLS-EU-Q16 (Spearman ρ: 0.35) and explained 26% of its total variance. Stratified analyses of the convergent validity between both instruments by age, sex, migration background, education level and chronic disease status showed moderate statistically significant correlations in all subgroups (range: 0.223 to 0.428). With an area under the curve of 0.66, the receiver operator curve indicated a satisfactory diagnostic performance of the SILS relative to the HLS-EU-Q16.

**Conclusions:**

The SILS provided an acceptable initial assessment of HL limitations among a heterogeneous population of mainly chronically ill patients in a primary care setting. With only one item, the SILS can be a short and effective tool for routine use in primary care and specialized care settings. Future research should test the SILS in other populations and pilot applications of the SILS in routine care.

## Introduction

Health literacy (HL) describes the ability to access, understand, appraise and apply (health-) information to make informed decisions regarding healthcare [1]. Low health literacy is a public health challenge worldwide. A recent meta-analysis estimated that low HL affects at least one third of the population in Europe [2]. Low HL levels are associated with reduced disease self-management skills, worse health outcomes, an increased number of hospitalizations, inadequate use of emergency rooms and less utilization of preventive services [3–6]. This association is well-described for chronically ill patients in a number of studies [7]. Diabetes patients with low HL, for example, have less knowledge of their disease, less frequently perform adequate self-management, have worse glycemic control and are less likely to achieve goal HbA1c levels compared to patients with adequate HL [8, 9]. Evidence is emerging that low HL contributes to inequalities in health [10, 11]. The limited ability to perform self-management in patients with low HL contributes to these inequalities, as self-management is increasingly important in chronic disease [12]. Together these facts highlight, the need to provide extra support to patients with low HL.

Two main approaches to support patients with low HL can be distinguished: an individual approach strengthening skills and abilities of the individual, and a systemic approach reducing demands and complexity of the healthcare system [13]. Both approaches benefit from adequate identification of patients with limited HL.

To measure HL, multiple instruments with different approaches, for different contexts and based on different theoretical frameworks have been developed in recent years [14, 15]. Several instruments have been validated for different languages and different populations. Some allow comprehensive multi-dimensional assessments while others are designed as screening instruments focusing on specific health-literacy related skills or abilities. So far, HL is mostly measured in research settings. Screening tools for clinical use are scarcely used in Germany and internationally [16], but their implementation in routine primary care and hospital settings is feasible as evidenced by US studies [17, 18].

In Germany, no validated brief screening tool is available for use by family doctors and other health care providers to determine their patients’ HL levels. The objective of this study was to test a previously developed single-item screener focusing on functional HL skills [19] among patients with diverse chronic diseases in a primary practice setting. Specifically, convergent validity of a single-item literacy screener (SILS) and the European Health Literacy Survey (HLS-EU-Q16) was examined. The HLS-EU-Q16 is the most frequently used instrument to measure HL in epidemiological and clinical studies in Germany. Due to its length, it is not feasible to use the HLS-EU-Q16 as brief screener. This study examined how closely the SILS relates to the HLS-EU-Q16, also in terms of diagnostic accuracy. Previous validation studies of HL instruments have often not provided consistent findings across subgroups of the study population [14]. Hence, we also investigated the influence of sociodemographic factors on convergent validity of the SILS and the HLS-EU-Q16.

## Methods

This study used cross-sectional data from a survey of 346 mainly chronically ill patients conducted between October 2015 and December 2017 in general practices in the German state North Rhine-Westphalia. General practices (*n* = 208) received an invitation to participate in the survey via fax. A member of the research team visited practices who expressed an interest to participate (*n* = 11, with *n* = 28 family doctors) to provide further information and enrol family doctors in the study. Family doctors recruited patients during regular consultations. Criteria for inclusion of patients were an age of at least 18 years, sufficient German language skills to fill out the survey and at least two practice visits during the past 12 months. Patients completed a paper-based survey in the practice after consultation with their doctor. Prior to this, written informed consent was obtained. The study was conducted in accordance with the Declaration of Helsinki and approved by the ethics committee of the University Hospital of Cologne (ID Number: 16–084).

### Measures

This article reports findings for study participants completing the HLS-EU-Q16, the SILS and a set of demographic questions. Previous findings from the larger patient survey, which included external HL ratings completed by the patients’ family doctors are reported elsewhere [20]. The HLS-EU-Q16 builds on a conceptual model of health literacy derived from a systematic literature review [21]. It measures four health literacy skills (i.e. accessing, understanding, appraising, and applying health information) in three domains (i.e. healthcare, disease prevention and health promotion). The present study uses the HLS-EU-Q16, a short version of the original survey consisting of 16 items. On a 4-point scale raging from “very easy” to “very difficult” the survey measures how easy it is for respondents to perform different activities [22–24]. It has acceptable psychometric properties and its sum score correlates highly with the sum score of the long version (*r* = 0.82) [24]. Based on the sum score three levels of health literacy are distinguished: inadequate (score <  = 8), problematic (score > 8 and <  = 12), and adequate (score > 12) [25].

The SILS is derived from a 16-item HL screener, originally developed by Chew et al. [26] and subsequently reduced to a 3-item instrument. Based on the 3-item instrument Morris et al. [19] developed a single item screener to identify patients, who have difficulty reading health related materials. The ability to read health related materials is a central component of the health literacy concept and more specifically of functional health literacy skills [27]. The SILS asks the question “How often do you need to have someone help you when you read instructions, pamphlets, or other written material from your doctor or pharmacy?”. Respondents answer on a five-point scale consisting of the following categories: never (1), rarely (2), sometimes (3), often (4) and always (5). The original English version was translated into German following the protocol of the European Social Survey for translation of questionnaires [15]. Two independent translators and an expert in health services research each translated it from English into German. In a consensus-meeting lead by an internationally experienced researcher a final version was developed [28].

The SILS has been validated in clinical studies against other HL instruments, including the Short Test of Functional Health Literacy in Adults (S-TOFHLA) and the newest vital sign (NVS) [19, 29, 30]. The SILS had a moderate to good association with these instruments [19, 29, 30]. In a study by Brice et al. [29] the SILS showed a similar ability to predict the S-TOFHLA as the 3-item screener developed by Chew et al. [26] and an alternative two-item screener.

### Statistical analyses

Descriptive statistics were used to analyse the sample’s demographic characteristics and their HL-levels based on the SILS and the HLS-EU-Q16. We examined convergent validity by exploring the association between the HLS-EU-Q16 and the SILS using Spearman’s correlation coefficients. Bootstrapping was used to calculate 95% confidence intervals (CIs) for the correlation coefficients. To explore the influence of the SILS on the explained variance in the HLS-EU-Q16 two-step multiple linear regression analyses were conducted adjusting for a priori selected covariates (i.e. age, sex, education, migration background, employment and chronic diseases). The possible impact of socio-demographic variables on the convergent validity between the SILS and the HLS-EU-Q16 was examined using stratified analyses. To assess the diagnostic performance of the SILS as compared to the HLS-EU-Q16, we calculated the sensitivity, specificity and likelihood ratios with CIs. A sum score of < 13 on the HLS-EU-Q16 was chosen as “gold standard” for limited HL in these calculations. This definition of limited HL includes both respondents with inadequate and with problematic HL levels. Receiver operator curves (ROC) were created to determine an adequate cut-off value for the SILS. Areas under the curve (AUC) were calculated to determine the predictive accuracy of the SILS. The level of significance was pre-set at alpha < 0.05 for all analyses. The analyses were conducted using IBM® SPSS® Statistics, version 27, and R [31].

## Results

Completed surveys (*n* = 346) were returned from 14 family doctors in 8 practices. Characteristics of the participating family doctors and practices are presented in Table 1.

**Table 1:** Characteristics of participating family doctors and practices

**Family doctors** (*n* = 14)	**n**	**%**
Sex		
Male	9	64.3
Female	5	
**Participating practices (*n* = 8)**		
Number of patients enrolled^a^		
≥ 500–1000	1	14.3
> 1000–1500	3	42.9
> 1500–2000	2	28.6
> 2000	1	14.3
Type		
Single practice	3	37.5
Group practice	5	62.5
Location		
Metro	6	75.0
Urban	2	25.0

^a^Data for one practice is missing

From the 346 patients included in the data set, 293 patients provided valid answers on the SILS and on ≥ 14 items of the HLS-EU-Q16 allowing to calculate a sum score. Table 2 summarizes patient characteristics of the study sample (*n* = 293) which includes patients between 20 and 89 years of age (Mean = 57.0, SD = 16.0).

**Table 2:** Sample characteristics

Characteristics	n		%	
Sex^a^				
Female	172		58.7	
Male	114		38.9	
Age^b^				
≤ 60 years	148		50.5	
> 60 years	133		45.4	
Employment status^c^				
Not working	135		46.1	
Working	148		50.5	
Education level^d,*^				
Low	113		38.6	
Moderate	77		26.3	
High	96		32.8	
Migration background^e^				
No	213		72.7	
Yes	69		23.5	
Number of chronic diseases^f^				
None	15		5.1	
1	85		29.0	
2	83		28.3	
3 or more	109		37.2	
Type of chronic disease^f^				
Cardiovascular disease	152		51.9	
Back pain	132		45.1	
Depression or other mental health disorders Mental disorders	79		27.0	
Diabetes	71		24.2	
Chronic obstructive pulmonary disease	48		16.4	
Rheumatism	32		10.9	
Cancer	28		9.6	
Stroke	12		4.1	
Chronic kidney disease	6		2.0	
Other	94		32.1	
HLS-EU-Q16**				
Inadequate HL	42		14.3	
Problematic HL	96		32.8	
Adequate HL	155		52.9	
	**Mean **	**SD**	**Min**	**Max**
SILS	4.2	1.0	1	5
HLS-EU-Q16 Score* (Cronbachs α = 0.92)	12.2	3.3	1	16

*Education level was defined as low = at most primary school, moderate = secondary school, high = university (of applied sciences) entrance qualification; **the overall HLS-EU-Q16 score was computed as the simple sum score of the 16 binary items; Missing data (n): ^a^7, ^b^12, ^c^10, ^d^7, ^e^11, ^f^1

According to the HLS-EU-Q16, almost half of the patients in our study (47.1%) have inadequate or problematic HL levels. Stratified analyses (data not shown) indicate that patients with employment and higher education levels had significantly higher self-reported HL levels (*p* = 0.016; *p* = 0.003). No significant differences in self-reported HL by age, sex and migration background were identified. Assessment of the convergent validity of the SILS and the HLS-EU-Q16 demonstrates a statistically significant positive correlation between the SILS and the HLS-EU-Q16 (Spearman ρ: 0.35; CI [0.229; 0.449]; *p* < 0.001).

Table 3 presents results of the stepwise linear regression model, which further investigates the relationship between the SILS and the HLS-EU-Q16. The findings show a significant improvement in explained variance from step 1 to step 2. Inclusion of the SILS in a model with socio-demographic variables and chronic disease status only (step 1), led to a significant increase in explained variance (R^2^change = 18%) and a total explained variance of 26% of the HLS-EU-Q16. Education level and employment status were the only variables with a unique individual impact on the HLS-EU-Q16 score after including the SILS in the model.

**Table 3:** Stepwise linear regression to predict the HLS-EU-Q16 score

Model summary	R^2^	R^2^ change
Step 1^a^	0.08	
Step 2	0.26	0.18
**Independent variables** (Full model, step 2)	**ß**	***p***
Age	0.783	ns
Sex	0.101	ns
Education level	0.447	≤ 0.025
Migration background	0.212	ns
Employment status	0.852	≤ 0.025
Chronic diseases^b^	0.339	ns
SILS	1.425	≤ 0.001

^a^Step 1: Socio-demographic variables and chronic diseases entered as a block, Step 2: SILS entered

^b^The variable ‘chronic diseases’ was entered as dichotomous variable (0 vs. 1 or more). All other variables were entered as displayed in Table 2

Stratified analyses of the convergent validity between the SILS and the HLS-EU-Q16 by age, sex, migration background, education level and chronic disease status showed statistically significant correlations in all subgroups. The Spearman correlations were all moderate and ranged from 0.223 to 0.428.

**Fig. 1:**
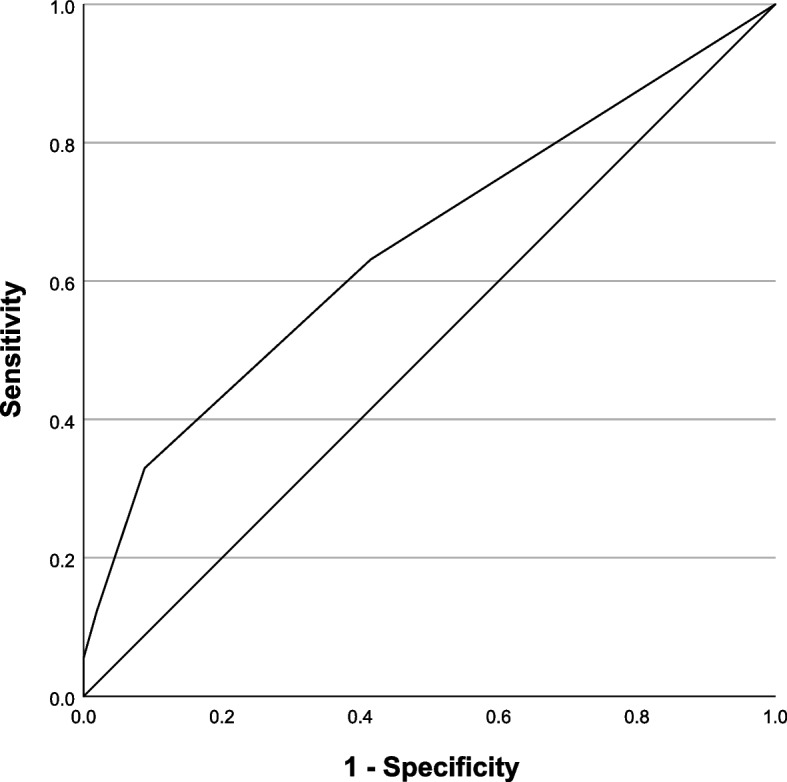
Receiver operator characteristics (ROC) curve for the SILS relative to the HLS-EU-Q16

Figure 1 displays the Receiver operator (ROC) curve of the SILS relative to the HLS-EU-Q16. The AUC was 0.66 (SE: 0.661; *p* < 0.001; CI [0.559; 0.724], indicating a satisfactory diagnostic performance of the SILS.

Table 4 presents findings regarding different possible threshold values for the SILS to indicate a positive test result (i.e. patients with limited HL level). A screening threshold of ≥ 2 optimized both sensitivity (66%) and specificity (58%) for the SILS and performed best regarding the likelihood ratios. When applying this threshold 53.2% (*n* = 156) of respondents are categorized with HL limitations.

**Table 4:** Diagnostic performance of the SILS in identifying patients with limited HL

**Threshold**	**Response category**^a^	**Sensitivity (95% CI)**	**Specificity (95% CI)**	**LR + (95% CI)**	**LR- (95% CI)**
≥ 1	Never	1.000 (-)	0.000 (-)	-	-
≥ 2	Rarely	0.659 (0.577–0.733)	0.581 (0.502–0.655)	1.572 (1.265–1.978)	0.587 (0.440–0.759)
≥ 3	Sometimes	0.326 (0.254–0.408)	0.910 (0.854–0.945)	3.610 (2.141–6.867)	0.741 (0.647–0.835)
≥ 4	Often	0.116 (0.073–0.180)	0.981 (0.945–0.993)	5.990 (2.109–Inf)	0.902 (0.838–0.955)
≥ 5	Always	0.058 (0.030–0.110)	1.000 (0.976–1.000)	Inf (2.096–Inf)	0.942 (0.898–0.981)

*LR* + positive likelihood ratio, *LR-* negative likelihood ratio

^a^Responses refer to the following question “How often do you need to have someone help you when you read instructions, pamphlets, or other written material from your doctor or pharmacy”?

## Discussion

This study examined the validity of a single item screener to determine patients’ HL levels in primary care practice settings in North Rhine-Westphalia, Germany. The screener demonstrated the ability to predict limited HL levels of patients as defined by the HLS-EU-Q16 with reasonably high sensitivity (66%) and specificity (58%). The diagnostic performance of the screener was satisfactory with an AUC of 0.66. Based on the ROC analyses a screening threshold of ≥ 2 represents the most optimal cut-off value for identifying limited HL levels using the single item screener. The ability of the single item screener to predict limited HL levels was similar for patients differing in age, sex, education, migration background, employment status and whether or not they suffer from chronic disease.

### Strengths

To our knowledge this is the first study validating a single-item HL screener against the HLS-EU-Q16. The HLS- EU-Q16 is a HL measure frequently used in Germany and internationally [32]. It is based on a multidimensional definition of HL and represents a more comprehensive, HL assessment tool than the S-TOFHLA or the NVS, which measure functional health literacy only [33]. The S-TOFHLA and the NVS have been used as criterion (or: gold standard) in previous validation studies of single-item HL screeners [19, 29, 30]. The diagnostic accuracy of the SILS in our study (AUC 0.66) compares to the diagnostic accuracy of the SILS reported in previous studies relative to the S-TOFHLA among hemodialysis patients (AUC 0.67) [29] and diabetes patients in the US (AUC 0.73) [19]. A study among a diverse patient population in Italy reported a higher diagnostic accuracy relative to the NVS (AUC 0.87) [30]. Two studies examining the validity of a similar single item screener among native Spanish speaking patients in the US found a lower diagnostic accuracy compared to our study, both with respect to the NVS (AUC 0.47) and the S-TOFHLA (AUC 0.32) [34, 35]. In sum, in comparison to existing evidence, the single-item screener in our study performs acceptable in identifying patients with limited HL. The similar performance for subgroups of patients suggests suitability of the screener for application among different patient populations.

### Limitations

This study tested the SILS among mainly chronically ill patients in family doctors’ practices in Germany. More research is required to validate the SILS among other populations (e.g. younger patients and patients without chronic disease) and in other health care settings (e.g. specialized care or paediatric settings). Future research should also examine reliability over repeated measures and consider the acceptability of HL measurement using the SILS from the perspective of patients. The single item screener validated in this study can only provide an initial indication of possible health literacy limitations in patients. It does not replace a detailed HL assessment to understand patients’ capability in multiple HL domains. Health literacy involves more than being able to read instructions, pamphlets, or other written material [36]. It also involves the ability to critically judge health information and resources, the ability to interact and express needs for health promotion [36], and encompasses psychosocial variables such as motivation [37]. Understanding these abilities requires more detailed screening approaches, for example using multiple items and stimuli to assess different aspects of HL skills or combining both self-report measures with objective performance-based HL assessments.

### Implications for clinical practice

This is the first study validating a brief HL screener for use in German health care settings. The screener can provide family doctors and other providers with a first indication that their patients have limited HL. It does not provide information on specific HL domains in which patients require support. Comprehensive tools allowing more detailed HL assessments in clinical settings are currently not available [38]. In the absence of such tools the SILS offers a useful way to increase providers’ awareness of potential HL limitations of their patients. Providers could incorporate the SILS in existing forms patients complete in writing at enrolment. This could contribute to sensitizing practice staff regarding additional support needs before patients consult with their doctor. As a consequence, practice staff could take additional measures to ensure that patients have a good understanding of the subject discussed during the consultation (e.g. by using teach-back methods) and by encouraging patients to ask questions.

Application of the SILS should not replace necessary supports for all patients however, such as the use of plain language, provision of accessible, evidence-based information materials, the initiation of additional support in navigating care processes (e.g. through coordinated care) and for self- management (e.g. through training and peer-support). Training healthcare providers in using plain language should be the first step to make healthcare systems more user friendly [39].

## Conclusion

The SILS provided an acceptable initial assessment of HL limitations among a heterogeneous population of mainly chronically ill patients in family practices in Germany. With only one item, the SILS can be a concise and effective tool to identify patients with additional support needs in routine primary care and specialized care settings. Future studies should pilot the SILS for specific and tailored applications in routine care contexts. This may include exploring patient preferences for different modes of administration of the SILS (e.g. written vs. oral administration and administration by different providers) as well as developing and testing education measures to support doctors and other practice staff, who apply the SILS in routine care settings. Education measures should focus on sensitizing staff regarding the HL concept and train them in HL-sensitive communication strategies.

## Data Availability

The datasets generated and/or analysed during the current study are not publicly available due to their containing information that could compromise the privacy of research participants. However, summarised data are available upon reasonable request from Arim Shukri.
